# Influences and Training Strategies for Effective Object Detection in Challenging Environments Using YOLO NAS-L

**DOI:** 10.3390/s26010190

**Published:** 2025-12-27

**Authors:** Gerald Steindl, Arnold Baca, Philipp Kornfeind

**Affiliations:** Department of Biomechanics, Kinesiology and Computer Science in Sport, Centre for Sport Science and University Sports, 1150 Vienna, Austria; arnold.baca@univie.ac.at (A.B.); philipp.kornfeind@univie.ac.at (P.K.)

**Keywords:** YOLO NAS-L, mean Average Precision (mAP) value, F1-score, precision and recall, training strategies, hyperparameters, batch size, batch accumulation, pre-trained weights, data augmentation

## Abstract

YOLO (You Only Look Once) is a one-stage detector that predicts object classes and bounding boxes in a single pass without an explicit region proposal step. In contrast, two-stage detectors first generate candidate regions. The YOLO NAS-L model is specifically designed to improve the detection of small objects. The purpose of this study is to systematically investigate the influence of dataset characteristics, training strategies and hyperparameter selection on the performance of YOLO NAS-L in a challenging object detection scenario: detecting swimmers in aquatic environments. Using both the mean Average Precision value (mAP)—which reflects the model’s global precision–recall performance and the F1-score, indicating the model’s effectiveness under realistic operating conditions—as evaluation metrics, this study investigates the effects of batch size, batch accumulation, number of training epochs, image resolution, pre-trained weights, and data augmentation. Our findings indicate that while batch size and image resolution had limited impact on performance parameters, the use of batch accumulation, pre-trained weights and careful tuning of training epochs were critical for optimizing model performance. The results highlight the practical significance of combining optimized hyperparameters, training strategies, and pre-trained weights to efficiently develop high-performing YOLO NAS-L models.

## 1. Introduction

Object detection is a branch of computer vision that teaches machines to understand visual content. It is an increasingly popular field within Deep Learning (DL) and has applications in various fields such as autonomous vehicles, robotics, augmented reality, medical fields, security systems and in sports, including recent approaches that explore contextual and multimodal object understanding [1,2,3]. For example, OMEGA [4], the company responsible for timekeeping for the international swimming federation WORLD AQUATICS, uses four cameras placed on the side of the pool to detect swimmers and measure their position and speed in real time. The YOLO (You Only Look Once) algorithm has been widely recognized for object detection since its inception in 2015 [5]. While the fourth version was released in 2020, version 10 became available in 2024. Unlike earlier approaches that utilized sliding windows followed by a classifier—which had to be executed hundreds or thousands of times per image—or methods that split the detection task into two steps (first identifying possible object regions and then applying a classifier), YOLO enables the detection task in a single pass through the network [6]. Jiang et al. [7] describe the rapid progress and widespread application of YOLO, attributing its popularity to the algorithm’s simple architecture, high computational efficiency, and ability to perform real-time object detection in videos. They also mention that YOLO has a strong generalization ability, as YOLO can learn highly generalized features that can be transferred to other domains, but the recognition accuracy should still be improved, especially for objects that look very similar and are close to each other. The YOLO NAS-L model, published in 2023, specializes in the detection of small objects and is designed to improve localization accuracy, enhance the performance-per-compute ratio, making it suitable for real-time edge-device applications [6,8]. This means that the YOLO NAS-L model is sufficiently resource-efficient and fast to perform real-time object detection directly on a decentralized device.

The NAS-L model variant offers developers a higher mean Average Precision (mAP) compared to the NAS-S and NAS-M, albeit with longer Inference Time, while YOLO NAS-L provides a lower mAP but with reduced Inference Time (Table 1). Inference Time is the time to process an image and make predictions and is usually measured in milliseconds [9]. The choice of the object detection model should therefore be tailored to the specific requirements [10].

Object detection models utilize pre-trained weights and fine-tuning on existing datasets such as Microsoft’s Common Objects in Context (COCO) dataset, aiming to improve accuracy by leveraging a large and diverse collection of images containing various objects across multiple scenarios. While persons are one of the 80 object classes included in COCO, swimmers are not. Also, many studies [11,12,13] show that it is very complicated to analyze the swimming technique only by visual object detection or to distinguish between sinking persons and swimming persons due to the external environment, the vibrational noise of the water surface and the uneven reflection of light.

In the individual stages, from dataset creation to model training, there are a lot of different influences and also possible strategies whose effects on each other and ultimately on the mAP value of the customized model need to be analyzed in such a difficult environment. In outdoor sports, there will hardly ever be the same image backgrounds, and athletes’ clothing will hardly ever be the same either. Achieving an increase in the mAP value solely through a larger image dataset does not seem to be the right solution.

## 2. Methods

**Figure 1:**
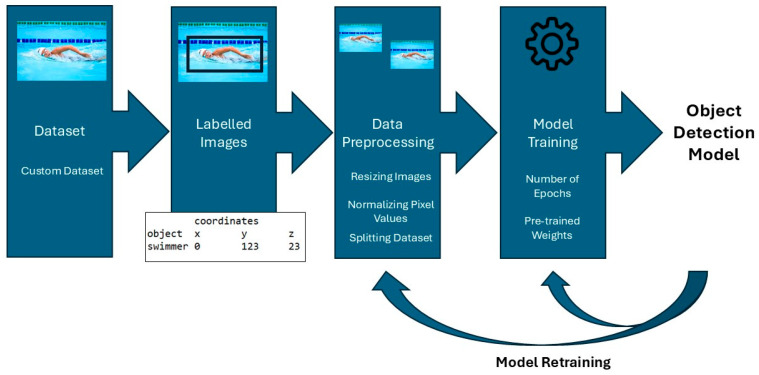
Steps towards an object detection model with various influences.

A total of 1500 images were acquired using a DJI Mavic 4 Pro drone (DJI, Shenzhen, China) under consistent weather conditions with direct sunlight. The images were captured above a lake with calm water and included two swimmers wearing distinct types of clothing. Data were collected from multiple viewing angles (frontal, dorsal, and lateral perspectives) at an approximate flight altitude of 5 m. The drone’s main camera is a wide-angle Hasselblad camera (Hasselblad AB, Gothenburg, Sweden) with a 4/3-inch CMOS sensor, a shutter speed of 1/10,000 s and a maximum photo resolution of 12,288 × 8192 pixels. In addition, over several days and at different times of day, 1041 images were recorded in two indoor swimming pools from various camera positions using a Luxonis OAK-D Lite depth-sensing camera (Luxonis Holding d.o.o., Ljubljana, Slovenia). Nearly all images—both from open-water and indoor environments—exhibit strong surface-water reflections, caused either by sunlight outdoors or by artificial lighting indoors. In many cases, the swimmers appear as small, low-resolution objects within the overall image frame, adding to the visual complexity of the dataset. A total of 2541 images were divided into three equally sized training, test and validation sets during model training. Images were recorded at a resolution of 4032 × 2268 pixels and subsequently annotated using the GitHub repository “LabelImg” (HumanSignal Inc., San Francisco, CA, USA), version 1.8.1 (S1). Necessary JSON files were generated from YOLO (TXT) formats using the GitHub repository “autoAnnoter” (naseemap47, GitHub; software version 3.0) (S2). An initial version of the training pipeline was inspired by an open-source repository, “YOLO-NAS” (naseemap47, GitHub; version 1.0) (S3). For the analysis of different training strategies during model training, the experiments were conducted using the “SuperGradients” framework (Deci AI Ltd., Tel Aviv, Israel), version 3.1.3 (S4) with CUDA Toolkit version 12.6 (NVIDIA Corporation, Santa Clara, CA, USA) support and PyTorch version 1.12.1 (PyTorch Foundation, Menlo Park, CA, USA) (S5), running continuously within a Docker environment using Docker Engine (Docker Inc., Palo Alto, CA, USA) via Docker Desktop version 4.52.0 on a Windows 10 operating system (Microsoft Corporation, Redmond, WA, USA) machine equipped with a CUDA-compatible NVIDIA GeForce RTX 4080 Super GPU (NVIDIA Corporation, Santa Clara, CA, USA) with 16 GB of dedicated memory (Figure 1).

To ensure a controlled and reproducible examination of the relationship between batch size, batch accumulation and detection accuracy, this study applies established training strategies for large-scale optimization. In particular, the linear learning–rate scaling rule proposed by Goyal et al. [14] is adopted, in which the base learning rate increases proportionally to the effective batch size in order to maintain stable gradient magnitudes during optimization. The effective batch size is defined as the product of the batch size and the batch accumulation. The learning rate ($\eta_{ew})$ is scaled proportionally to the change in effective batch size relative to the baseline configuration:

(1) $$\eta_{ew}=\eta_{0}\times s$$

(2) $$s=\frac{B_{eff}}{B_{0}\times A_{0}}$$

The baseline configuration $B_{0}=16$ and $A_{0}=1$ was chosen to match the standard training regime of the employed model. This setup represents the middle batch size without gradient accumulation on the available GPU hardware and therefore serves as a robust, hardware-aligned reference point. The initial learning rate $\eta_{0}=5\cdot 10^{-4}$ corresponds to the default hyperparameter used by the “SuperGradients” YOLO-NAS training framework (S3).

Furthermore, a dynamic warmup is employed to prevent instability in the early training phase, a technique that has proven essential in large batch size training regimes such as Peng et al. [15] and You et al. [16].

(3) $$\eta_{warmup}=\frac{\eta_{ew}}{1000}$$

Instead of setting an arbitrary number of training epochs or predefined training limits, we used a fully data-driven approach to identify the transition from the learning phase to the convergence plateau. This transition was formalized as a change point (CP) in the performance curve. Mathematically, a change point τ is defined as the epoch at which the rate of change exhibits a distributional shift.

(4) ΔmAP(e)=mAP(e)−mAP(e−1)

The Pruned Exact Linear Time (PELT) algorithm estimates this point by minimizing the penalized cost function, where l(⋅) denotes the likelihood and β is a penalty controlling model complexity.

(5) $$C(\tau )=\sum_{i=1}^{\tau }l(x_{i}|\theta_{1})+\sum_{i=\tau +1}^{T}l(x_{i}|\theta_{2})+\beta$$

The resulting structural break indicates the onset of the plateau phase.

A series of inferential statistical procedures was applied to analyze the data. These included one-way and two-way analyses of variance (ANOVA), supplemented by Tukey HSD post hoc comparisons to identify significant group differences; linear mixed-effects models (MixedLM) to account for potential within-group dependencies; multivariate analyses of variance (MANOVA) to evaluate effects across multiple dependent variables simultaneously; and several breakpoint detection techniques (PELT, binary segmentation, and segmented regression) to identify plateau points or structural changes in the learning curves. All statistical analyses were performed in Python version 3.9 (Python Software Foundation, Wilmington, DE, USA) using the “statsmodels” package (statsmodels Development Team; software version 0.14.5) (S6).

## 3. Results

**Figure 2:**
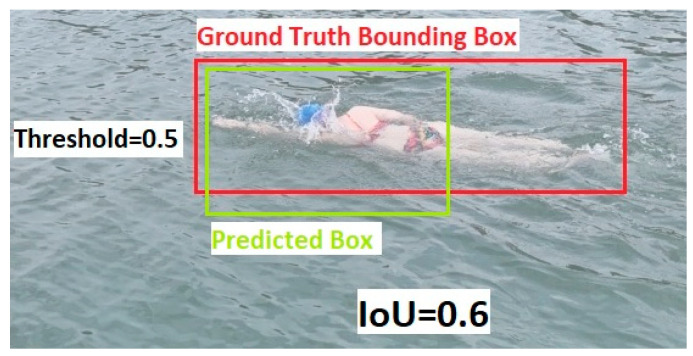
Example of correct object recognition when the IoU threshold is 0.5.

**Figure 3:**
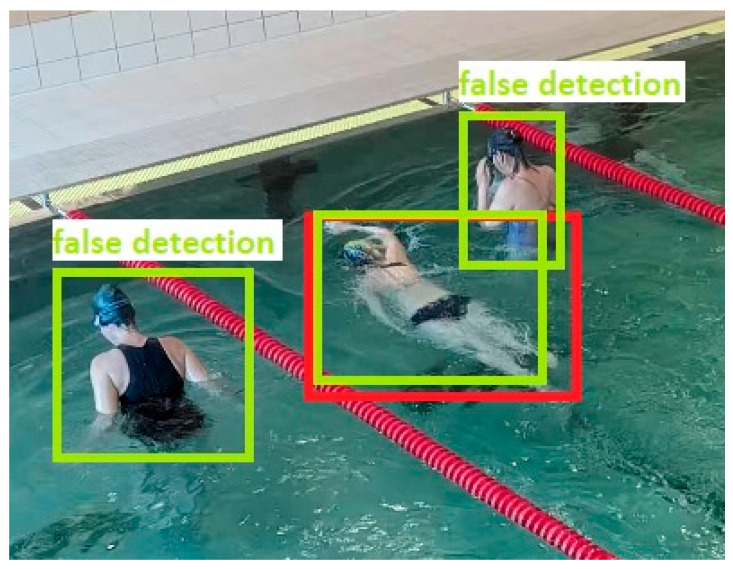
Example of false object detection.

To comprehensively evaluate the performance of the respective models, mAP@50 is used as a central metric. This value represents the mean Average Precision at an IoU threshold of 0.5, meaning that an object (in this study: the swimmer) is considered correctly detected if there is at least 50% overlap between the predicted bounding box and the manually annotated ground truth bounding box, see Figure 2 and Figure 3.

The mAP@50 metric is derived from the area under the Precision–Recall (PR) curve, which plots precision (the proportion of correct detections among all detections) against recall (the proportion of correctly detected objects among all existing objects) across varying confidence thresholds.

**Figure 4:**
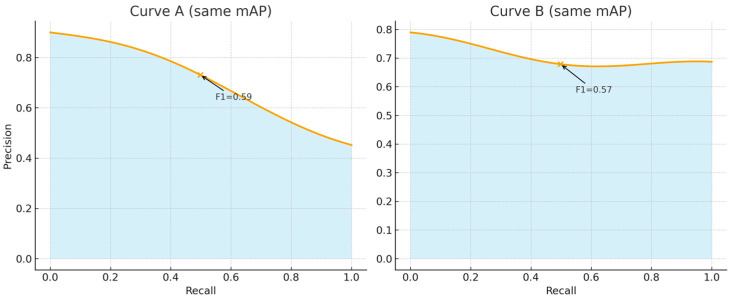
Two different PR curves with the same mAP value but different F1 scores.

Since mAP summarizes the complete PR curve, it provides a global performance measure, reflecting how well a model performs across its entire range of operating points rather than at a single, fixed threshold, see Figure 4.

In addition to mAP@50, the F1 score at an IoU threshold of 0.5 (F1@50) is employed as a complementary metric to capture a model’s behavior at a specific decision point. Unlike mAP@50, which integrates over all thresholds, the F1@50 metric reflects the local performance of the model at a precise threshold setting that may be operationally relevant (for instance, the confidence level used during inference). This makes F1@50 particularly useful for assessing how reliably a model performs under practical deployment conditions, where detections are accepted or rejected based on a predetermined confidence value. A higher F1@50 value indicates a more favorable compromise, characterized by both high precision and high recall. Taken together, these two metrics offer a more holistic understanding of model performance. While mAP@50 provides a global view of the detection quality across all confidence levels, F1@50 delivers a threshold-specific view of precision–recall balance. Using both measures allows for a deeper characterization of a model’s strengths and weaknesses, supporting informed decisions when comparing models or optimizing detection settings.

### 3.1. Batch Size and Batch Accumulation

Training models in the field of computer vision typically requires substantial memory resources. The batch size refers to the number of images processed by the model in a single training iteration. Ultralytics [17], the developer of various YOLO models (including YOLOv5 through YOLOv8 and YOLOv12), describes batch size as a critical hyperparameter that significantly influences the training dynamics, resource utilization, and ultimately, the performance of the final model.

To conduct training exclusively on the GPU while accounting for memory limitations, the technique of batch accumulation can be employed. Batch accumulation is intended to increase the effective stack size, thereby reducing noise in the mAP@50 value—an effect that can be particularly valuable when GPU memory is limited. For example, with a batch accumulation factor of 4, the model’s weights are updated only after every fourth iteration within a training epoch. The number of iterations per epoch is calculated by dividing the number of training images by the batch size. With the hardware resources available to us (a Windows 10 machine equipped with a CUDA-compatible NVIDIA GPU with 16 GB of dedicated memory), it was only possible to train images at a resolution of 414 × 414 pixels with a maximum batch size of 32 without using batch accumulation. Training with a larger batch size or higher image resolution would have required more dedicated GPU memory. To nevertheless analyze comparable effective batch sizes, these settings were approximated using batch accumulation (e.g., batch size = 16, batch accumulate = 4 → effective batch = 64). This approach is well established for simulating large-batch training under memory constraints and is fully documented as part of the experimental design. Since the resulting dataset is unbalanced, with missing factor combinations, statistical analysis was conducted using a linear mixed-effects model (MixedLM). This model supports incomplete designs and includes random intercepts per run (run_id) to account for run-to-run variability. Fixed-effects tests evaluated the influence of batch size, batch accumulation, and their interaction. Interaction plots and effect batch size estimates were additionally included to robustly assess their impact on mAP@0.50 and F1@0.50. The experiments followed a factorial design with two independent variables: Batch size (16, 32) and batch accumulation (1, 2, 4). This resulted in a total of five experimental configurations (due to the partially crossed design). Each configuration was repeated with eight different training seeds to capture stochastic variability in the optimization process, yielding a dataset of 40 training runs. Each seed was applied across all configurations, forming a crossed experimental structure that enables the specification of a random intercept per seed in the mixed-effects model. The dependent variables were mAP@0.50 and F1@0.50, recorded at the end of each training run. Missing outcomes (e.g., due to non-convergent runs) were treated as missing data within the statistical model.

**Figure 5:**
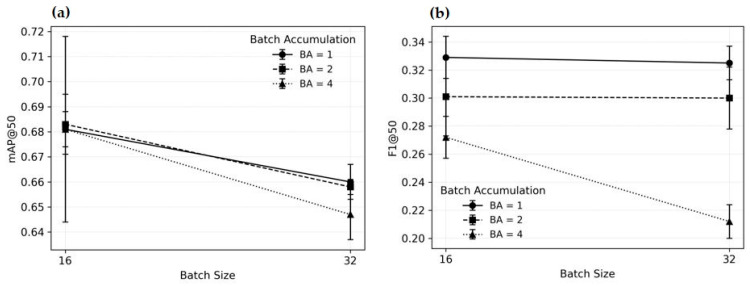
Interaction plot: Effects of batch size and batch accumulation (BA) on (**a**) the mAP@50 and (**b**) the F1@50.

**Figure 6:**
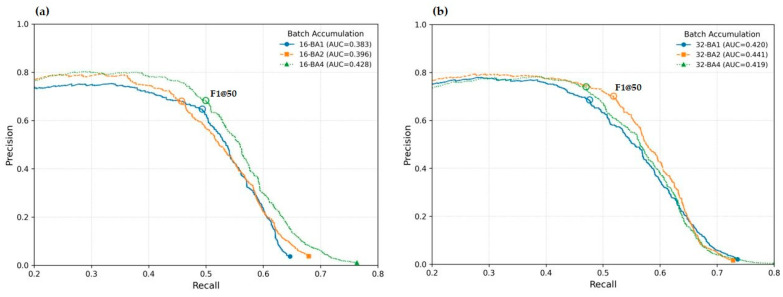
Precision–recall curves at IoU = 0.50 with (**a**) a batch size of 16, (**b**) a batch size of 32 and different batch accumulation factors. Each curve corresponds to a single, randomly selected training run from the set of repeated experiments.

The mixed-effects model revealed no statistically significant main effects of batch size, batch accumulation, or their interaction on mAP@50 value (Table 2, Figure 5a and Figure 6). Batch size 32 showed only a small, non-significant downward trend relative to batch size 16 (β = −0.021, *p* = 0.074). Batch accumulation values of 2 and 4 did not produce measurable changes in mAP@50 (*p* > 0.90). Likewise, none of the interaction terms approached statistical significance.

The mAP@50 appears robust with respect to the examined hyperparameters. Neither increasing the effective batch size nor using higher gradient accumulation meaningfully affects detection accuracy.

In contrast, the F1 metric showed a more pronounced sensitivity to Batch accumulation (Table 3, Figure 6). Batch accumulation 4 led to a statistically significant decrease in F1@0.50 (β = −0.058, *p* < 0.001). Batch accumulation 2 showed a marginal negative trend (β = −0.027, *p* = 0.076). Batch Size alone did not exhibit a significant main effect (*p* = 0.796).

However, a significant interaction between Batch Size 32 and Batch accumulation 4 was observed (β = −0.055, *p* = 0.010), indicating that the negative effect of Batch accumulation 4 on F1 becomes more pronounced when the batch size is increased.

While mAP@50 value remains stable across hyperparameter settings, F1@0.50 deteriorates significantly when batch accumulation is increased to 4, particularly in combination with batch size 32 (Figure 6b). This suggests that the precision–recall balance is more sensitive to large effective batch sizes than the overall detection accuracy.

### 3.2. Number of Epochs

According to Ultralytics [18], the image size used during the model training step can influence the model accuracy. Based on the estimated plateau metrics (median mAP@50 and F1@50 beyond the breakpoint), we conducted a Multivariate Analysis of Variance (MANOVA) to evaluate the joint influence of image size on both performance criteria. Let

(6) $$Y=(\begin{array}{c} plateau\text{\_}mAP50\\ plateau\text{\_}F1 \end{array}).$$

The multivariate model is formulated as

(7) Y=α+B⋅image_size+ε,

where B encodes the size-dependent effects. MANOVA tests whether the multivariate centroids of the plateau metrics differ significantly across input resolutions, thus capturing both accuracy and stabilization dynamics within a unified statistical framework. Across six runs, the plateau epoch falls between Epoch 37 and 66, yielding average plateau performance of mAP@50 ≈ 0.4675, F1@50 ≈ 0.3383, and Area Under the Curve (AUC) ≈ 76.57 (Table 4).

The MANOVA indicates no significant multivariate effect of image size on (plateau-mAP50, plateau-F1) (Wilks’ λ = 0.3165; F = 3.24; *p* = 0.178) (Table 5). Exploratory univariate ANOVAs likewise show non-significant effects for mAP@50 (F = 3.43; *p* = 0.168) and F1@50 (F = 6.28; *p* = 0.085), with a suggestive trend for F1@50. By image size, 1024 × 1024 px exhibits the highest mean mAP@50 (~0.475) and AUC (~78.03), whereas 640 × 640 px yields the highest mean F1@50 (~0.379). The three breakpoint methods (PELT, Binseg, segmented regression) disagree substantially (e.g., segmented BP: 2–100), highlighting sensitivity to data structure/parameter choices; bootstrap estimates (mean 150, SD 0) are non-informative here—likely due to resampling that disrupts temporal dependence and pushes the algorithm to the terminal index, suggesting block bootstrap as a better alternative.

Overall, results suggest similar plateau performance across image sizes with mild advantages for 1024 × 1024 px (mAP@50/AUC) and 640 × 640 px (F1@50).

### 3.3. Data Size & Runtime

**Figure 7:**
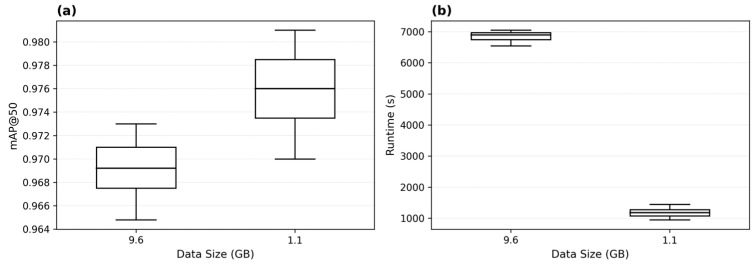
Box plots: Effect of training data size on (**a**) detection performance measured by mAP@50 and (**b**) computational runtime in seconds.

Prior to training a YOLO model, input images are typically resized by the model—depending on the configuration—into a fixed image dimension, which is usually square, with few exceptions. Ultralytics [18] states that the image size used during training affects both model accuracy and computational load. As previously demonstrated, no highly significant effect of the image resolution used during training on the mAP@50 and F1@50 could be observed in our case. The attempt to reduce the resolution of the 1500 drone images—captured under consistent weather conditions—from 4032 × 2268 pixels to 1280 × 720 pixels during data preprocessing, which decreased the dataset size from 10.2 GB to 1.71 GB (–83.2%), had no significant effect on overall model performance (F(1, 2) = 0.889, *p* = 0.445) (Table 6, Figure 7). However, this reduction in data size had a highly significant impact on runtime performance (F(1, 2) = 224.77, *p* = 0.004) (Table 7, Figure 7).

However, the two-way ANOVA conducted on the expanded dataset, which included an additional 1041 contrasting images from a completely different domain, revealed a significantly higher mAP@50 (Table 8). The highly significant main effect of data size therefore reflects not merely the number of images but the substantive expansion of data representation. This phenomenon is well documented: performance improvements arise primarily from increased diversity rather than from a mere increase in volume [19,20].

**Figure 8:**
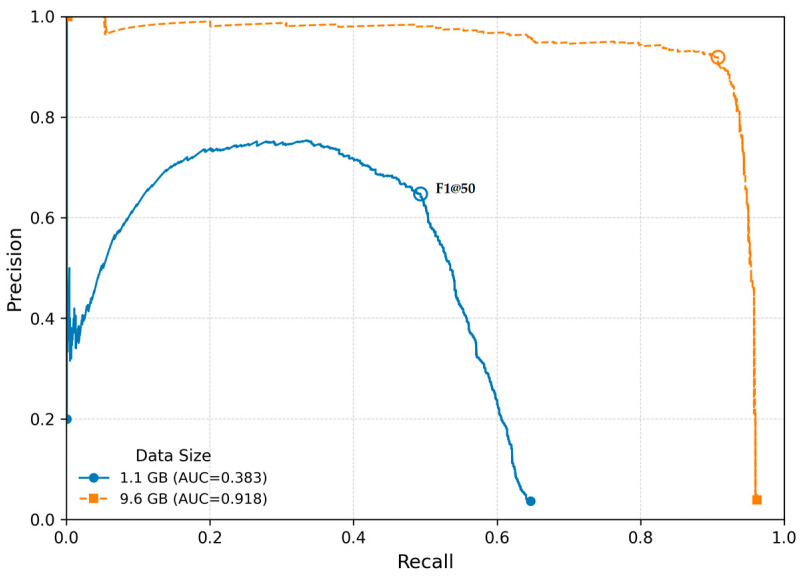
Precision–recall curves at IoU = 0.50 with a batch size of 16 for two different dataset sizes. Each curve corresponds to a single, randomly selected training run from the set of repeated experiments.

Across all conditions, models achieved significantly higher mAP@50 compared to F1@50, F(1, 8) = 2634.36, *p* = 2.30 × 10^−11^ (Table 8, Figure 8). Both metrics improved significantly with increasing dataset size, *F*(1, 8) = 1731.94, *p* = 1.22 × 10^−10^. The interaction between dataset size and metric was not significant, *F*(1, 8) = 3.37, *p* = 0.104, indicating that mAP@50 and F1@50 benefit similarly from the larger dataset. When analyzed separately, mAP@50 increases from 0.6845 (1.71 GB) to 0.9810 (10.2 GB) and F1@50 increases from 0.3467 (1.71 GB) to 0.6182 (10.2 GB). Both metrics therefore show substantial performance improvements, with mAP@50 consistently producing higher values across all conditions.

Tukey HSD post hoc comparison showed that all pairwise contrasts between the metric–dataset combinations were statistically significant (all *p* < 0.05). Specifically, models trained on the larger dataset (10.2 GB) exhibited significantly higher mAP@50 as well as F1@50 compared to models trained on the 1.71 GB dataset (Table 9). Additionally, the comparisons between mAP@50 and F1@50 within each dataset size confirmed that mAP@50 values were consistently and significantly higher than F1 scores at IoU = 0.50, reflecting the inherently different scales of the two metrics.

Overall, these results show that image downscaling in the data preprocessing step leads to a significant deterioration in model performance and that the two metrics consistently represent different performance scales. The absence of significant interaction suggests that both metrics benefit similarly from the enlarged training dataset.

### 3.4. Pre-Trained Weights

The developer of YOLO-NAS models, Deci AI, provides pre-trained model parameters—referred to as pre-trained weights—via its “SuperGradients” library (S5). These weights have been obtained using images from Microsoft’s Common Objects in Context (COCO) dataset. They can either be used directly for object detection if the objects of interest are already classified within the COCO dataset, or for fine-tuning additional models by leveraging generalized features, such as the shape of human bodies for human detection tasks. Models trained with COCO weights achieved substantially higher mAP@50 scores than models trained without pretrained weights (0.6845 vs. 0.5753). This difference was statistically highly significant (*p* < 0.000001). These results indicate that COCO pretraining provides a strong advantage in detection accuracy at IoU = 0.50. The Tukey HSD test confirmed significant differences between all groups (Table 10).

**Figure 9:**
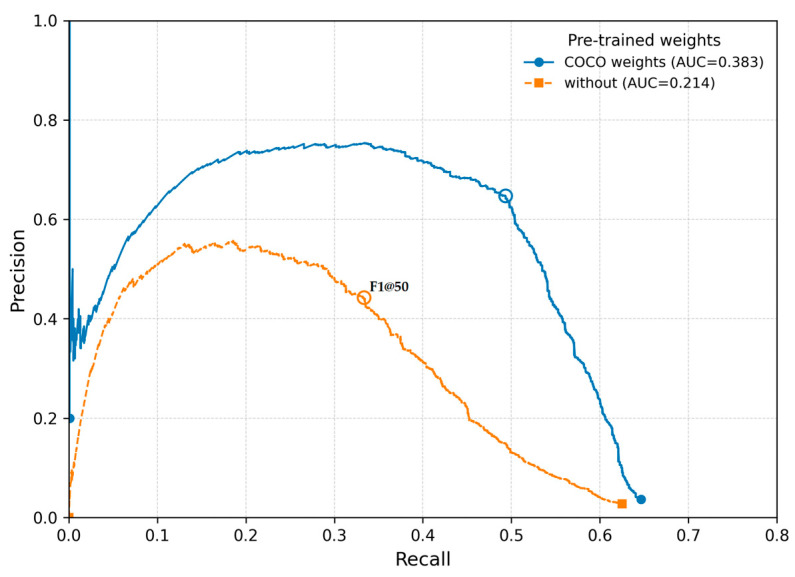
Precision–recall curves at IoU = 0.50 with a batch size of 16 for the effects of pre-trained weights. Each curve corresponds to a single, randomly selected training run from the set of repeated experiments.

A pronounced improvement was also observed for F1@50 when COCO weights were used (0.3467 vs. 0.1749), and this effect was likewise statistically significant (*p* < 0.000001). The two metrics differ strongly from each other (*p* < 10^−10^) (Table 11, Figure 9). As expected, mAP@50 values are systematically higher than F1@50 values because mAP is more stable and less sensitive to individual classification errors. The significant interaction effect (*p* = 0.0026) indicates that the relative improvement gained from COCO weights is larger for F1@50 than for mAP@50—COCO contributes disproportionately more to the balance between precision and recall.

### 3.5. Data Augmentation

**Figure 10:**
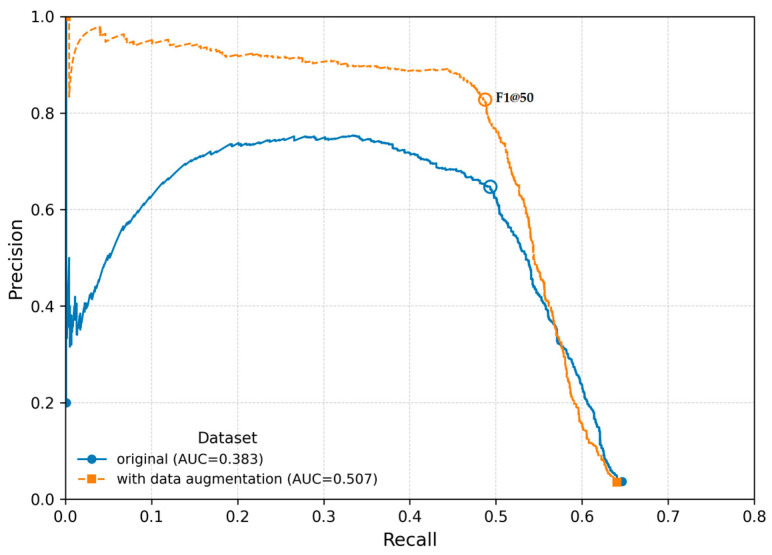
Precision–recall curves at IoU = 0.50 with a batch size of 16 for the effects of data augmentation. Each curve corresponds to a single, randomly selected training run from the set of repeated experiments.

In the field of Deep Learning (DL), data augmentation is a technique used to increase the size of a dataset by generating artificial variations in existing images, thereby improving the model’s generalizability [21]. According to Kaur et al. [21], data augmentation techniques can be categorized into the following groups: methods based on geometric transformations, color modifications, random occlusions, and deep-learning-based approaches. In our study, we applied the first three categories. Using the GitHub repository “imgaug” (S7), images were rotated, flipped, backgrounds were altered, objects were pixelated, resized, and contrast adjustments were applied. Following Zoph’s recommendation [22], we expanded the number of images used for model training by adding 2000 augmented images, while keeping the test and validation datasets unchanged to avoid unrealistic evaluation results. The two-way ANOVA revealed a significant main effect of dataset size on model performance (F(1,4) = 25.87, *p* = 0.007), as well as a highly significant effect of the metric (F(1,4) = 1324.65, *p* < 0.001) (Table 12). The interaction between dataset size and metric was also significant (F(1,4) = 21.30, *p* = 0.0099), indicating that the impact of dataset size depends on the metric considered. Post hoc analyses (Tukey HSD) showed that the larger dataset significantly improved F1@50 scores (*p* = 0.0081), whereas no significant difference was observed for mAP@50 (*p* ≈ 0.99). Overall, these findings suggest that additional data (including data augmentation) primarily enhanced F1@50, while having little effect on mAP@50 (Figure 10).

## 4. Discussion

The influence of training strategies, such as data augmentation and hyperparameters, such as the number of training epochs, on the performance of YOLO models is widely acknowledged from both a theoretical [10,17,21] and practical [23] perspective. The broad range of application areas for object detection models today—spanning from large-scale objects in the traffic sector to extremely small entities in satellite or microscopic medical imaging—combined with the increasing variety of available YOLO model versions, raises the important question of which specific YOLO version (e.g., YOLOv8, YOLOv11, YOLOv12, YOLO NAS S, etc.) should be selected for a given task.

According to Terven et al. [6], YOLO NAS models were designed to improve the detection of small objects, with a focus on enhancing localization accuracy and optimizing the performance-per-compute ratio, making them particularly well-suited for real-time applications on edge devices. As a subsequent project of ours aims to track positional changes in swimmers in real time, we chose to utilize the YOLO NAS L model. However, from an applied perspective, we observed that the YOLO NAS L model, released in 2023, still exhibits significant compatibility issues with essential supporting libraries and computing platforms, such as CUDA. Whether other YOLO variants might offer better compatibility or be more appropriate for future project requirements was not explored within the scope of this study. Interestingly, our research revealed that swimmers have not been classified as distinct object categories in any major datasets—such as Microsoft’s Common Objects in Context (COCO), which serves as the basis for the pre-trained weights used in YOLO NAS L. This insight is relevant for interpreting our results. Given the broad applicability of object detection models, it would be both interesting and necessary to extend our findings to objects that are already categorized in such datasets. The broader availability of image data for these objects would also allow for experimentation on more powerful computing systems.

While Liu et al. [24] already provide promising initial approaches for improving the efficiency and performance of multimodal object detection models, the present work establishes foundational insights into the optimization of models based on single-modal RGB datasets, with substantial further research needed in both domains.

Analysis of the test data suggests that batch accumulation can facilitate training of object detection models on less powerful hardware. However, unlike mAP@50, F1@50 appears more sensitive to batch accumulation (Table 3, Figure 5 and Figure 6). Consequently, after the initial assessment of its impact on model performance, batch accumulation was omitted in subsequent experiments, and a batch size of 16 was adopted—except for the epoch hyperparameter analysis, where hardware constraints necessitated a batch size of 4.

The MANOVA results regarding image size indicate only marginal advantages: 1024 × 1024 pixels yielded slightly higher mAP@50 and AUC values, while 640 × 640 pixels showed a minor edge for F1@50. These differences, however, were not statistically significant (Table 5). Thus, the hypothesis that larger image sizes consistently improve performance cannot be confirmed. The observed trends suggest that image size within the tested range plays a limited role. It is preferable to avoid high batch accumulation values and to use moderate image sizes for training. Future research should therefore prioritize other influential factors, such as data augmentation, since image size alone does not lead to substantial performance gains.

Based on these findings, we strongly recommend avoiding any reduction in image resolution during preprocessing—even in challenging environments where runtime optimization is desired (Table 8 and Table 9, Figure 8). Our analyses, supported by prior studies [3,8], consistently demonstrate that models benefit most from larger datasets that increase variance, diversity, and complexity of training examples.

Furthermore, the use of pre-trained model parameters proved to have a highly significant effect on mAP@50 and F1@50 (Table 10 and Table 11, Figure 9). Consequently, when training a YOLO NAS L model, the use of pre-trained weights should not be omitted—even if, as in our case, no images of swimmers are included in the COCO dataset.

The impact of data augmentation requires careful consideration. Our analysis indicates that additional data, including augmented images, primarily enhance classification accuracy (F1@50), while localization accuracy (mAP@50) remains largely unaffected (Table 12). Therefore, data augmentation should not be viewed as a universal solution but applied selectively and in moderation. Augmented images should be placed only in the training folder to ensure realistic evaluation of generalization. Furthermore, image transformations should be mild, preserving object integrity and bounding box quality. This aligns with best practices outlined by Zoph [22] and is consistent with prior studies [25], indicating that augmentation can enhance the robustness of object detection models. At the same time, these works emphasize that augmentation should be applied cautiously and with only mild image transformations to preserve object integrity. Future research should focus on optimized, domain-specific augmentation strategies that target improvements across both metrics.

In light of the currently limited amount of scientific literature that addresses the impact of training strategies and hyperparameter selection on model performance, this article may serve as a foundational contribution and provide motivation for further research in this field.

## 5. Conclusions

This scientific study systematically analyzed the influence of training strategies and hyperparameter choices on the performance of a YOLO NAS L object detection model, providing a pipeline-level ablation of commonly adjusted parameters. Beyond confirming the robustness of mAP@50 across a wide range of configurations, the results highlight the importance of complementary metrics such as F1@50, which proved more sensitive to practical training decisions, particularly batch accumulation. The findings demonstrate that certain widely assumed optimization strategies—such as increasing batch accumulation or image resolution—do not necessarily lead to measurable performance gains, while others, including the use of pre-trained weights and carefully designed data augmentation, have a statistically significant impact. By combining rigorous statistical modeling with detailed precision–recall analysis, this work contributes actionable guidelines for training YOLO NAS models under realistic computational constraints. As such, the study provides both methodological insight and practical recommendations, serving as a foundational reference for future research on training optimization in modern object detection frameworks.

## Figures and Tables

**Table 1:** Relationship of the three YOLO-NAS models S, M, L to the mAP value and the Inference Time [10].

Model	mAP	Inference Time [ms]
YOLO NAS-S	47.5	3.21
YOLO NAS-M	51.55	5.85
YOLO NAS-L	52.22	7.87

**Table 2:** MixedLM results for the effects of batch size and batch accumulation on mAP@50 at an image size of 414 × 414 pixels.

Predictor	Coefficient (β)	Std. Error	z	*p*
Intercept	0.681	0.008	80.48	<0.0001
Batch Size (32)	−0.021	0.012	−1.79	0.074
Accumulation (2)	0.001	0.012	0.12	0.902
Accumulation (4)	0.000	0.012	0.02	0.983
Effective Batch Size (64 = 32 × 2)	−0.004	0.017	−0.21	0.832
Effective Batch Size (128 = 32 × 4)	−0.013	0.017	−0.79	0.432

**Table 3:** MixedLM results for the effects of batch size and batch accumulation on the F1@50 at an image size of 414 × 414 pixels.

Predictor	Coefficient (β)	Std. Error	z	*p*
Intercept	0.329	0.011	30.51	<0.0001
Batch Size (32)	−0.004	0.015	−0.26	0.796
Accumulation (2)	−0.027	0.015	−1.77	0.076
Accumulation (4)	−0.058	0.015	−3.78	<0.001
Effective Batch Size (64 = 32 × 2)	0.002	0.022	0.11	0.917
Effective Batch Size (128 = 32 × 4)	−0.055	0.022	−2.57	0.010

**Table 4:** Per-Run Results with an effective batch size of 16.

Image Size	Run	Epoch Plateau	Breakpoint (PELT-Algorithm)
416 × 416 px	1	45	65
416 × 416 px	2	37	35
640 × 640 px	1	66	50
640 × 640 px	2	46	55
1024 × 1024 px	1	44	50
1024 × 1024 px	2	56	75

**Table 5:** Results of the MANOVA.

Effect	Test	Value	DF
Intercept	Wilks Lambda	0.0007	2
	Pillai’s Trace	0.9993	2
	Hotelling-Lawley	1509.5409	2
	Roy’s Greatest Root	1509.5409	2
Image size	Wilks Lambda	0.3165	2
	Pillai’s Trace	0.6835	2

**Table 6:** One-way ANOVA results for the effects of data size (9.91 GB and 1.10 GB) on mAP@50 with a batch size of 16 and an image size of 416 × 416 px.

	Sum of Squares	df	F	*p*-Value
Data Size	0.000044	1	0.88898	0.44528
Residual	0.000098	2		

**Table 7:** One-way ANOVA results for the effects of data size (9.91 GB and 1.10 GB) on the runtime with a batch size of 16 and an image size of 416 × 416 px.

	Sum of Squares	df	F	*p*-Value
Data Size	31,472,100	1	224.774223	0.004419
Residual	28,003	2		

**Table 8:** Two-way ANOVA results for the effects of data size (10.2 GB and 1.71 GB) on mAP@50 and F1@50 with a batch size of 16, batch accumulation of 1 and a training image size of 416 × 416 px.

Effect	Df	Sum of Squares	F	*p*-Value
Dataset size (1.71 GB vs. 10.2 GB)	1	0.2420	1731.94	$1.22\times 10^{-10}$
Metric (mAP@50 vs. F1@50)	1	0.3681	2634.36	$2.30\times 10^{-11}$
Interaction (Dataset × Metric)	1	0.00047	3.37	0.104
Residual	8	0.00112	-	-

**Table 9:** Tukey HSD post hoc comparisons between the four metric–dataset combinations.

Comparison	Mean Difference	Adjusted *p*-Value
F1@50 (1.71 GB)–mAP@50 (1.71 GB)	0.3378	<0.001
F1@50 (1.71 GB)–F1@50 (1.71 GB)	0.2715	<0.001
F1@50 (1.71 GB)–mAP@50 (10.2 GB)	0.6343	<0.001
mAP@50 (1.71 GB)–F1@50 (10.2 GB)	−0.0663	0.0006
mAP@50 (1.71 GB)–mAP@50 (10.2 GB)	0.2966	<0.001
F1@50 (10.2 GB)–mAP@50 (10.2 GB)	0.3628	<0.001

**Table 10:** Tukey HSD post hoc comparisons for all pairwise group contrasts (Weights/without weights × Metric).

Comparison	Mean Difference	95% CI	*p*-Value
COCO–mAP@50 vs. COCO–F1@50	+0.3378	[0.3048, 0.3707]	<0.001
COCO–F1@50 vs. without–F1@50	−0.1718	[−0.2047, −0.1388]	<0.001
COCO–F1@50 vs. without–mAP@50	+0.2286	[0.1956, 0.2616]	<0.001
COCO–mAP@50 vs. without–F1@50	–0.5095	[–0.5425, –0.4766]	<0.001
COCO–mAP@50 vs. without–mAP@50	–0.1092	[–0.1421, –0.0762]	<0.001
without–F1@50 vs. without–mAP@50	+0.4004	[0.3674, 0.4333]	<0.001

**Table 11:** Two-way ANOVA results for the effects of pre-trained weights on mAP@50 and F1@50 with a batch size of 16, batch accumulation of 1 and a training image size of 416 × 416 px.

Effect	Df	Sum of Squares	F	*p*-Value
Weights	1	0.05919	372.40	$5.39\times 10^{-8}$
Metric	1	0.40863	2570.83	$2.54\times 10^{-11}$
Weights × Metric	1	0.00294	18.49	0.0026
Residual	8	0.00127	-	-

**Table 12:** Two-way ANOVA results for the effects of dataset size on mAP@50 and F1@50 with a batch size of 16, batch accumulation of 1 and a training image size of 416 × 416 px.

Effect	Df	Sum of Squares	F	*p*-Value
Dataset size	1	0.005957	25.87	0.007050
Metric	1	0.305020	1324.65	0.000003
Dataset size × Metric	1	0.004905	21.30	0.009914
Residual	4	0.000921	-	-

## Data Availability

The data used in this study are available upon request from the corresponding author.

## Supplementary Materials

The following supporting information can be downloaded at (accessed on 12 July 2025): https://github.com/aleju/imgaug, GitHub repository S1: “LabelImg”. https://github.com/naseemap47/autoAnnoter, GitHub repository S2: “autoAnnoter”. https://github.com/naseemap47/YOLO-NAS, GitHub repository S3: “YOLO-NAS”. https://pypi.org/project/super-gradients/3.1.3/, Python-Library S4: “Super-Gradients”. https://pytorch.org/get-started/previous-versions/, S5: PyTorch. https://pypi.org/project/statsmodels/, Python-Package S6: “Statsmodels”. https://github.com/aleju/imgaug, GitHub repository S7: “imgaug”.

## Abbreviations

The following abbreviations are used in this manuscript:

AUC	Area Under the Curve
BA	Batch Accumulation
COCO	Microsoft Common Objects
CUDA	Compute Unified Device Architecture
DL	Deep Learning
MANOVA	Multivariate Analyses Of Variance
mAP	Mean Average Precision
MixedLM	Linear Mixed-Effects Model
PELT algorithm	Pruned Exact Linear Time (PELT) algorithm
YOLO	You Only Look Once
